# Fertilization rate and its determinants in intracytoplasmic sperm injection

**DOI:** 10.12669/pjms.321.8329

**Published:** 2016

**Authors:** Shireen Jawed, Rehana Rehman, Mohammad Ashfaq Ali, Umme Hani Abdullah, Hina Gul

**Affiliations:** 1 Dr. Shireen Jawed, M.Phil Physiology, Assistant Professor of Physiology, Islam Medical & Dental College, Sialkot, Pakistan; 2 Dr. Rehana Rehman, PhD Physiology, Assistant Professor of Physiology, Aga Khan University, Karachi, Pakistan; 3 Dr. Mohammad Ashfaq Ali, M.Phil Bio Chemistry, Assistant Professor, Liaquat National Medical College, Karachi, Pakistan; 4 Umme Hani Abdullah, Medical student, Aga Khan University, Karachi, Pakistan; 5 Hina Gul, Senior Research Analyst, Prontolinks International (Pvt) Ltd, Lahore, Pakistan

**Keywords:** Intracytoplasmic sperm injection, Fertilization, Fertilization rate, Interleukin

## Abstract

**Objective:**

To identify predictors of fertilization rate in patients of unexplained infertility after intracytoplasmic sperm injection (ICSI).

**Methods:**

Retrospective analysis of females (282) enrolled in quasi experimental design for ICSI at “Islamabad Clinic Serving Infertile Couples” was carried out from July 2013 till June 2014. Females with unexplained infertility were included, whereas well defined male and female causes of infertility were excluded. Fertilization rate (FR) was calculated as percentage transformation of micro injected oocytes into two pronuclei. Categorical variable of FR defined on the basis of 50% FR grouped females; Group I with FR ≤50% and Group II with FR >50%. The groups were compared in terms of demographic variables, base line hormones and oocyte parameters. Univariate logistic regression was executed to obtain odds ratio with 95% confidence interval to quantify the association of predictors like age, duration of infertility, oocytes parameters, hormones; Estradiol, progesterone, follicle stimulating hormone (FSH), luteinizing hormone, prolactin and cytokines interleukin-Iβ (IL-Iβ) with the FR.

**Results:**

In our study out of 282 females, 19 (6.73%) were in group I and 263 (93.26%) comprised of Group II. Females with high FR(group II) had low Progesterone and FSH (p=0.04, p=0.02) respectively. Mature oocytes (OR: 0.35; 95% CI 1 – 2.56) and IL-Iβ in follicular phase (OR: 1.04; 95% CI: 0.000- 1.20) were significant positive predictors of FR while peak progesterone and FSH had significant negative effect on it

**Conclusion:**

Fertilization of oocytes in females of unexplained infertility depended on maturity of oocytes and optimal amounts of ILI- β released by developing follicles in the follicular phase of stimulation cycles of ICSI.

## INTRODUCTION

Infertility is one of the major emerging problems associated with social issues, anxiety, stress and psychological upset of infertile partners.^1^ The occurrence of infertility in Pakistan is higher (10-15%) as compared to 2.9% prevalence worldwide.^2^ Infertile couples seek assisted reproductive technologies (ART) especially intracytoplasmic sperm injection (ICSI) in hope of successful outcomes especially in cases of male infertility and failure after in vitro fertilization (IVF). The primary objective of all ART clinics is to achieve maximum implantation potential as well as minimize chances of multiple pregnancies.^3^-^5^ Even though greater numbers of infertile couples are eager, yet fearful for failure of implantation and treatment results.

Fertilization rate (FR) is percentage of transformation of micro injected oocytes into two pronuclei.^6^,^7^ The implantation rate (IR); the number of pregnancies per embryo transferred, has been reported to vary from 10% to 40% in different clinics.^8^,^9^ It is established that low FR results in poor IR and pregnancy outcome in comparison to patients with greater FR.^9^,^10^ Case control study conducted by Ou YC and his colleagues determined better clinical pregnancies in females who underwent ICSI because of better FR as compared to low FR acquired in IVF techniques.^11^ In addition to that, study conducted by MP Rosen et al. determined FR as a biological assay for IR.^7^

Failure of treatment after ICSI is attributed to a long list of factors which vary depending on the cause of infertility. In 25% of the cases, etiology of infertility cannot be found. This unexplained infertility is described with normal semen counts together with optimal ovulatory functions, presence of patent tubes and a normal uterine cavity.^12^ It has been observed that failure of fertilization and cleavage is more likely to occur in couples with unexplained infertility as compared to tubal factor infertility, and male sperm problems.^13^ Having agreed to FR as a strong predictor of IR, we aimed to identify biophysical and biochemical parameters that could affect FR in patients of unexplained infertility.

## METHODS

The retrospective analysis of data from a quasi-experimental design carried out in Islamabad Clinic Serving Infertile (June 2010 till August 2011) was done from July 2013 till June 2014. The study was carried after ethical approval from the review board of the clinic. For this analysis, couples with unexplained infertility for more than two years with female’s age ranging from 20 till 35 years were included. Infertility due to male factor and females with polycystic ovaries, endometriosis and endocrine abnormalities were excluded. Down regulation of ovaries was done by gonadotrophin releasing hormone agonist (Injection DecaPeptyl) from Day 21 of previous cycle. They had controlled ovarian stimulation (COS) by gonadotrophins (InjPuregon I/M or S/C) after which ovulation induction (OI) was planned on the basis of maturity of follicles. Venous sample was taken on OI day for estimation of peak serum estradiol (E2), Progesterone (P) and interleukin I-β (IL-Iβ). Oocytes retrieved after 36±1 hours were micro injected by spermatozoa in labeled microinjection dish on the heated stage of an inverted phase contrast microscope under 200 x magnifications. Normal fertilization was determined 16–20 hours after ICSI by presence of two pronuclei and FR estimated by number of fertilized oocytes per number of microinjected oocytes.^7^,^14^ Embryos were evaluated on alternate days and were transferred in the blastocysts stage. The implantation rate was number of gestational sacs observed on TVS divided by the number of embryos transferred.^15^

Peak E2 was estimated by using commercially available kit for Human E2 Enzyme Immunoassay Kit Cat. No. 07BC-1111 by MP Biomedicals, USA. Serum P was determined by using commercially available kit for Human Progesterone Enzyme immunoassay, Kit Cat No. KAP 1451 by Bio Source, Belgium. Peak IL-Iβ was determined by using commercially available kit for Human IL-Iβ-EASIA Enzyme Immunoassay Kit Cat. No. KAP 1211 by DIA source Immuno Assays, Luteal support after egg collection was maintained by P vaginal pessaries (Cyclogest 400 mg) twice a day started after OPU.

### Outcome measures

Serum ß human chorionic gonadotrophin (hCG) measurement was done 14 days after embryo transfer and clinical pregnancy was confirmed by presence of cardiac activity on transvaginal ultrasound scan(TVS ) two weeks afterwards. On the basis of beta hCG and TVS results were categorized into non pregnant with beta hCG <25units, and clinical pregnancy with beta hCG>25 and cardiac activity confirmed by TVS. The different reproductive rates were transformed into groups I and II on the basis of FR ≤ 50% and >50% respectively. Univariate logistic regression was executed to obtain odds ratio with 95% confidence interval to quantify this association of FR with the predictors.

### Statistical Analysis

Data was analyzed by SPSS (version 16; SPSS Inc., Chicago, IL, USA). The qualitative variables were represented by frequencies and percentages like (age group), mean ± SD for continuous/quantitative variables. Association between the dependent and independent variables were analyzed by binomial logistic regression analysis. Results were given as odd ratio and 95% confidence interval (CI) to evaluate the effect of each predictor on FR, p value <0.05 was considered statistically significant.

## RESULTS

Descriptive characteristics of study population are shown in Table-I. Females with FR>50% had high basal E2, luteinizing hormone and prolactin levels but the result was not significant. Group II had greater number of retrieved and mature oocytes (p=0.04, p=0.0001). There was no significant difference in peak E2 in groups whereas peak P was lower in this group (p=0.04). Table-II reveals that the Metaphase II (OR: 0.35; 95% CI 1 – 2.56 and Peak IL-Iβ (OR: 1.04; 95% CI: 0.000- 1.20) are significant positive predictors of FR, while peak P and FSH had significant negative effect on it.

**Table-I:** Comparison of cycle characteristics on the basis of fertilization rate.

Variables	Fertilization Rate ≤ 50% (*n*=19)	Fertilization Rate > 50% (*n*=263)	P value
*Demographic Variables*			
Age	31 ± 4.5	32 ± 4.6	0.26
Duration of infertility	8.5 ±3.9	7.5 ±3.8	0.33
Body Mass Index	24.2 ± 3.6	25.2± 2.6	0.06
*Base line Hormones*			
Estradiol before treatment(pg/ml)	171 ± 106.6	219 ± 149.9	0.18
Follicle stimulating hormone (IU/L)	6.65 ± 1.12	6.69 ± 1.08	0.02
Luteinizing Hormone (IU/L)	4.98 ± 1.39	5.21 ±1.43	0.46
Prolactin (µg/L)	21.8 ± 7.51	22.39 ± 5.5	0.6
*Oocyte Parameters*			
Preovulatory follicle count	8.05 ± 2.1	17.7 ± 1.8	0.05
Number of oocytes	7.11 ± 1.07	7.72 ±1.69	0.04
Number of mature oocytes	2.8± 1.40	7.4 ± 1.60	0.000
*Peak Hormone Levels*			
Peak Progesterone (ng/ml)	1.78 ±0.64	1.44 ±0.75	0.046
Peak estrogen p(g/ml)	2.25 ± 5.7	2.32± 3.01	0.49
Peak Interleukin I-β (pg/ml)	68.9±39.9	119±63.75	0.01

Values are expressed as Mean ± SD

**Table-II:** Predictors of fertilization rate.

Predictors of fertilization rate	Β	SE	Odd ratio (OR)	P value	95%CI
Age	- 0.026	0.052	0.97	0.6	0.001- 3.98
Duration of infertility	0.475	9.360	1.6	1.00	0.000 - 0.001
No of oocytes/patient	4.288	1.614	1.000	0.6	0.000 - 0.09
Metaphase II	21.927	2.654	0.35	0.00	1 - 2.56
Preovulatory follicle count	-11.62	1.44	0.999	.000	0.012 – 1.45
Peak IL-Iβ	0.044	85.5	1.04	0.031	0.000 – 8.97
Estradiol before treatment	- 0.001	0.002	0.999	0.648	0.0001- 8.56
Peak estradiol	- 0.008	15.52	0.99	1.0	0.00 -4.70
Peak Progesterone	- 6.125	1.370	1.000	0.002	0.000- 1.20
Follicle stimulating hormone	-1.354	0.245	0.258	0.000	0.000 -0.9866
LH	0.120	0.173	1.019	0. 40	0.00-1.5
Prolactin	0.018	0.042	1.019	0.60	0.000-1.4

Logistic linear regression to find predictors of fertilization rate

## DISCUSSION

Implantation of human embryo is a well co-ordinated sequenced event of apposition and adhesion of the invading blastocyst in the endometrial bed during the window of implantation.^16^ The sensitivity of antral follicles to gonadotropic drugs, response to stimulation, oocyte maturity, fertilization, embryo quality and endometrial thickness, all contribute to magnitude of COS and success rates of ART. In all procedures, failure of fertilization is the stage which stops the procedure and wipes the hopes and expectations of the infertile couples. The problem is intensified more in the couples to whom the reproductive endocrinologists have no true explanation of the cause of infertility.

During ICSI, number of oocytes estimated by TVS as preovulatory follicle count (PFC) is the strongest predictor of number of oocytes obtained during oocyte pick up.^17^ In our study although the number was high in group II, yet turned out to be a negative predictor of FR. Basal FSH (done on the day 2 or 3 of the cycle)although is considered as reflector of OR cannot predict ART outcome due to inter cyclic variability.^18^,^19^ We observed that it was a negative predictor of FR. E2 produced during follicular phase of female reproductive cycle improves endometrial receptivity for implantation of blastocyst.^17^ Good number of oocytes and high E2 measured on the day of hCG administration reflects response and pregnancy outcome that has been subject of debate by a number of researchers.^20^,^21^ In a previous study our group reported that females with higher peak E2 had greater number of retrieved, mature and fertilized oocytes with increased FR and IR.^16^ In this study, peak E2 turned out to be a negative predictor of FR in patients of unexplained infertility.

The P concentrations measured on the day of hCG administration were higher in females with decreased FR. It can be explained on the basis of its role on ovarian follicles, with adverse effects on oocyte maturation, fertilization or early cleavage.^22^ The premature luteinization that occurred in our study population decreased the pronuclei formation(FR). Our results are in accordance with a study by Hill et al. in which serum P levels were not proved to be markers of oocyte and embryo quality, and better fertilization rate.^23^

It is documented that fertilization of oocytes involves complex series of events requires growth, development and cytoplasmic maturation of oocytes microinjected with spermatozoa.^10^ The role of cytokines is well established for pre-implantation embryo development, protection of embryo, endometrium sustenance, successful implantation and positive pregnancy outcome by increase in expression of adhesive protein integrins at the implantation site in the endometrium.^24^ In our study, we observed that IL-I β took part in fertilization of oocytes. The association of IL-β with fertilization results is supported by Zollner et al. however they measured intrafollicular levels of this cytokine.^25^ With the existing knowledge of FR as predictor of implantation we may forecast results of implantation in unexplained infertility cases (failure of fertilization is the main reason of failure after ICSI) by looking into all variables that affect FR.

## CONCLUSION

The FR in females with unexplained infertility was not dependent on number of oocytes, basal FSH, LH and prolactin, neither was dependent on peakE2 and P hormone levels. IL-I β helped in events that caused fertilization of oocytes.

## RECOMMENDATIONS

Detection of intrafollicular cytokine profiles like IL-I β,-IL-8, IL-12 andIL-18 as prognostic markers for oocyte fertilization and embryo quality can help in prediction of success after ART.
